# Genomes From 117 Vertebrate Species Reveal Rapidly Evolving Segmental-Duplication Landscapes

**DOI:** 10.1093/gbe/evag043

**Published:** 2026-02-27

**Authors:** Alber Aqil, Saiful Islam, Faraz Hach, Ibrahim Numanagić, Naoki Masuda, Omer Gokcumen

**Affiliations:** Gilbert S. Omenn Department of Computational Medicine and Bioinformatics, University of Michigan, Ann Arbor, MI, USA; Department of Biological Sciences, State University of New York at Buffalo, Buffalo, NY, USA; Lewis-Sigler Institute for Integrative Genomics, Princeton University, Princeton, NJ, USA; Institute for Artificial Intelligence and Data Science, State University of New York at Buffalo, Buffalo, NY, USA; Vancouver Prostate Centre, Vancouver, British Columbia, Canada; Department of Urologic Sciences, University of British Columbia, Vancouver, British Columbia, Canada; Department of Computer Science, University of Victoria, Victoria, BC, Canada; Gilbert S. Omenn Department of Computational Medicine and Bioinformatics, University of Michigan, Ann Arbor, MI, USA; Institute for Artificial Intelligence and Data Science, State University of New York at Buffalo, Buffalo, NY, USA; Department of Mathematics, University of Michigan, Ann Arbor, MI, USA; Department of Mathematics, State University of New York at Buffalo, Buffalo, NY, USA; Center for Computational Social Science, Kobe University, Kobe, Japan; Department of Biological Sciences, State University of New York at Buffalo, Buffalo, NY, USA

**Keywords:** vertebrate evolution, segmental-duplication networks, biological networks, tandem duplication, interspersed duplication, platypus

## Abstract

Segmental duplications are major drivers of evolutionary innovation; yet, their dynamics across vertebrates remain poorly understood. Here, we identify segmental duplications from long-read-sequenced genomes of 117 vertebrates and one starfish, generating the largest multispecies dataset of its kind. We find that vertebrate genomes show a higher propensity for tandem duplications than for interspersed duplications. However, when focusing only on subtelomeric regions, avian and mammalian genomes show the opposite propensity toward interspersed duplications. We also observe that, across vertebrates, tandem duplications tend to be larger than interspersed duplications. Next, we construct a segmental-duplication network for each species and use network-derived metrics to quantify the duplication landscape for that species. We then compute interspecies distances for each metric and find that these distances show at most weak correlations with phylogenetic distance, indicating that segmental-duplication landscapes evolve rapidly. Functional-enrichment analysis of hyperduplicated genes reveals a strong enrichment in platypus for pheromone response, driven by the expansion of the vomeronasal pheromone receptor *V1R* gene family. Overall, our results uncover the general properties of vertebrate segmental duplications, demonstrate the lability of segmental-duplication landscapes, and highlight the utility of network-based approaches for studying genome evolution.

Significance Gene and regulatory region duplications are a fundamental source of evolutionary raw material. Here, we generate segmental-duplication calls from 117 vertebrate species. We find that vertebrate genomes have a bias toward tandem duplications relative to interspersed duplications. However, in the subtelomeric regions, birds and mammals exhibit an opposite bias toward interspersed duplications. Our analysis of segmental-duplication networks demonstrates that duplication landscapes evolve rapidly, following species-specific rather than phylogenetic patterns. These findings indicate that the genomic architecture underlying segmental duplications is highly dynamic, uniquely shaping each lineage's potential to adapt. Our study provides the most comprehensive view of vertebrate segmental duplications to date and establishes a network-based framework for studying genomic structural evolution.

## Introduction

Duplication of genes (Ohno 1970) and regulatory regions (Shew et al. 2025; Weaver and Lowe 2025; Morrissey et al. 2026) has been proposed to play a major role in vertebrate evolution. In particular, segmental duplications can create redundant paralogs of functional regions (Carroll 2005), allowing mutations that would have been deleterious in the ancestral copy to be tolerated. Such duplications can therefore circumvent valleys in the fitness landscape (Banse et al. 2024) and increase the probability of reaching higher adaptive peaks (Li and Zhang 2025) by enabling the co-option of duplicates for new functions (Innan and Kondrashov 2010; Cisneros et al. 2026). The mutation rate is elevated in segmental duplicates relative to single-copy regions (Vollger et al. 2023), further raising the likelihood that duplication will acquire new functions. Alternatively, duplication of genes or regulatory elements can drive dramatic changes in expression levels (Aqil et al. 2025), which may itself confer a fitness advantage.

Additionally, genomic regions rich in segmental duplications are prone to accumulating further duplications by increased rates of recombination errors (Kim et al. 2008; Lin and Gokcumen 2019), thereby affecting the potential for adaptation. Finally, as functional analyses extend to nonhuman species, identifying segmental duplications becomes important, since they can also generate spurious functional associations between genomic loci (Saha and Battle 2018; Aqil and Gokcumen 2025). For these reasons, identifying segmental duplications and studying the evolution of segmental-duplication landscapes across diverse species is imperative.

One way to study the evolution of segmental-duplication landscapes is through segmental-duplication networks. Previous work has shown that models involving gene duplication followed by divergence between duplicates yield realistic properties in biological networks (Solé et al. 2002; Pastor-Satorras et al. 2003; Vázquez et al. 2003; Lin and Zhang 2010; Lü and Wang 2020; Abdullaev et al. 2021, 2025). Although duplication-and-divergence network models were first proposed more than two decades ago (Solé et al. 2002; Pastor-Satorras et al. 2003; Vázquez et al. 2003), they have rarely been tested on large empirical datasets of segmental duplications (Bergman et al. 2006; Vollger et al. 2019; Abdullaev et al. 2021, 2025). Such empirical networks can reveal aspects of the segmental-duplication landscape, such as the presence of duplication clusters, which non-network approaches cannot capture.

**Fig. 1.**
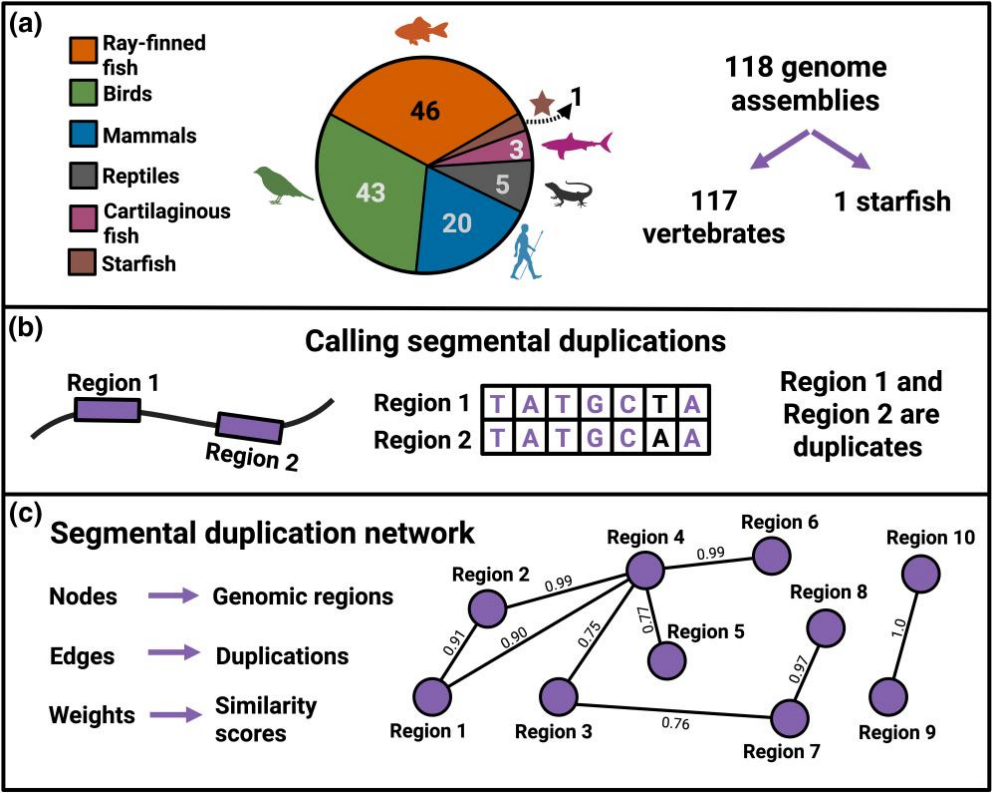
Methodological framework. a) The number of species in each taxonomic class in our data. b) A schematic showing segmental-duplication calling from genome sequences. The image on the left represents a chromosome with two genomic regions marked as Region 1 and Region 2. The middle image shows that the sequences from the two regions align with a high similarity score. Regions 1 and 2 are, therefore, duplicates. c) A schematic segmental-duplication network. For each species, we used 13 network properties and the average similarity score across duplications (a non-network property) to quantify the segmental-duplication landscape.

Here, we call segmental duplications genome-wide from 117 vertebrate species and one starfish using long-read-assembled genomes (Fig. 1a and b) generated by the Vertebrate Genomes Project (Formenti et al. 2026). This creates the largest multi-species catalog of segmental duplications to date. Using this dataset, we explore the properties of vertebrate segmental duplications and construct segmental-duplication networks to study their evolutionary dynamics (Fig. 1c).

Overall, our analyses of segmental-duplication networks offer new insights into the forces shaping genomic structural variation across vertebrates. By integrating large-scale comparative genomics with network approaches, we show how segmental-duplication landscapes evolve and drive species-specific adaptive potential.

## Results

### Segmental Duplications, Transposable Elements, or Ohnologs?

**Fig. 2.**
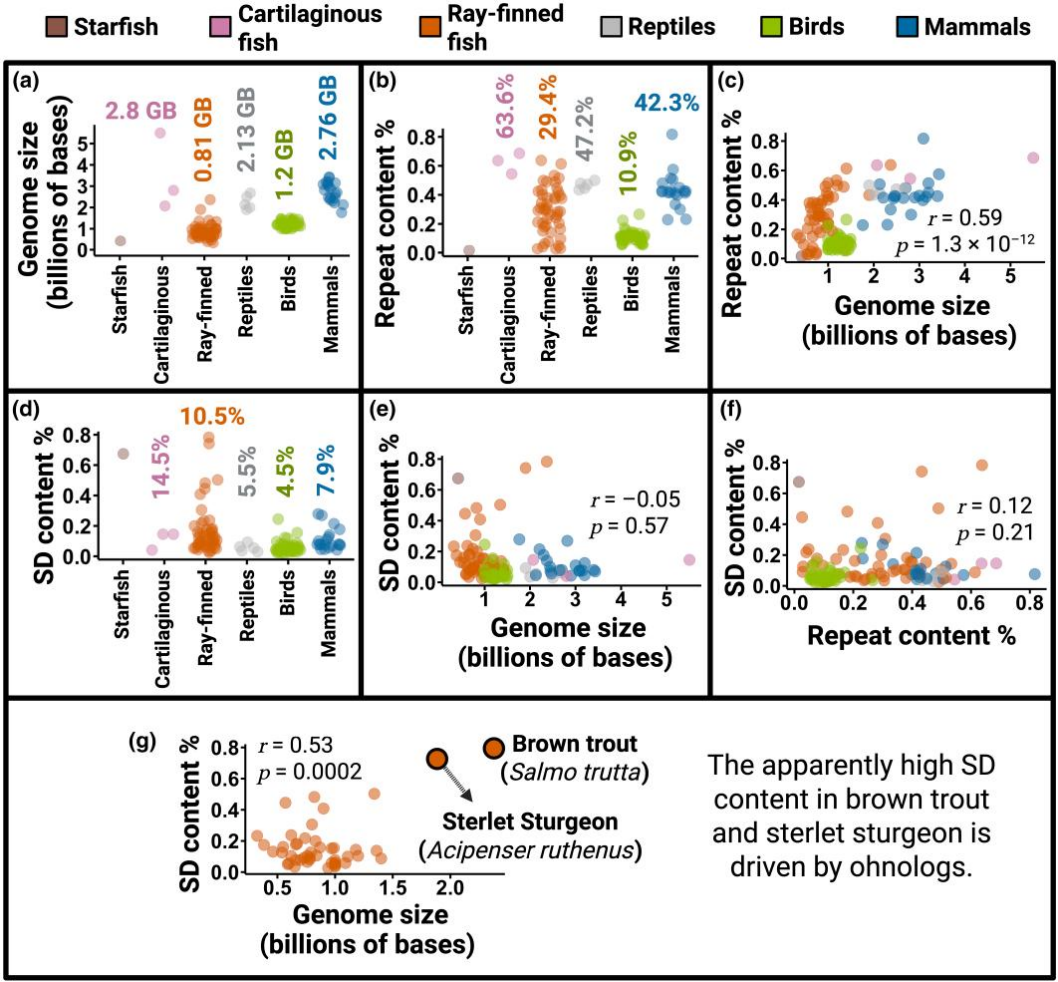
Segmental duplications, repeat content, and ohnologs. a) Distribution of genome size across species. The epaulette shark (*Hemiscyllium ocellatum*) is an outlier among our sample of vertebrates for its large genome size (>5 billion base pairs). The numbers shown are the medians for the corresponding taxonomic classes. We do not show the number for starfish because it is only one data point. b) Distribution of the percentage of the genome covered by repeats (transposable elements and simple repeats) across species. The blue whale (*Balaenoptera musculus*) is an outlier among mammals for its high repeat content. The percentages shown are the medians. c) Variation in repeat content (driven by transposable elements) explains variation in genome sizes. d) Percentage of the genome covered by segmental duplications (SDs) across species. The percentages shown are the medians. The median SD coverage calculation for ray-finned fish excluded the brown trout and sterlet sturgeon on account of high false-positive rates in SD detection due to ohnologs. e) Variation in segmental-duplication content across species does not explain genome-size variation. f) Repeat content and segmental-duplication content are uncorrelated across species. If inadequate repeat masking were systematically inflating segmental-duplication calls, we would have seen a negative correlation between repeat content and segmental-duplication content. g) The correlation between genome size and the fraction of the genome covered by duplications among ray-finned fish is driven by two outliers: the sterlet sturgeon and the brown trout (a salmonid). After removing these two species, the correlation coefficient drops to −0.05. The apparently high segmental-duplication content of brown trout and sterlet sturgeon actually represents a high ohnolog content.

In this study, we call segmental duplications using genomes from 117 vertebrate species and one starfish generated by the Vertebrate Genomes Project (Formenti et al. 2026). The sizes of these genomes are shown in Fig. 2a. Calculating genome-size variation within each taxonomic class with at least five representatives, we find that genome-size variation among ray-finned fish (coefficient of variation, CV = 0.41) is higher than that among reptiles (CV = 0.15), birds (CV = 0.13), and mammals (CV = 0.17). Moreover, partitioning the genome-size variance into within- and between-class components, we find that most of the genome-size variance (82%) is attributable to differences between, rather than within, taxonomic classes.

Our approach for detecting segmental duplications identifies regions of high sequence similarity (alignment span ≥ 900 bases; see Methods). Without prior filtering, this approach could theoretically capture three types of duplicated sequence: transposable elements, ohnologs (remnants from whole-genome duplications), and segmental duplications.

First, we address the possibility of transposable elements getting spuriously detected as segmental duplications. Transposable elements and short repeats are pervasive in vertebrate genomes (Elliott and Gregory 2015) and must therefore be systematically identified and masked prior to calling segmental duplications. To achieve this, we masked each genome with RepeatMasker using species-specific libraries wherever available. In particular, we used species-specific libraries for all mammals, all birds, all reptiles, 23 out of 46 ray-finned fish species, and 2 out of 3 cartilaginous fish species. For the remaining species, we used class-level repeat libraries. The fraction of each genome masked for transposable elements (and other repeats) across taxonomic classes is shown in Fig. 2b. Similar to the variation in genome sizes, ray-finned fish genomes show greater variation in repeat content (coefficient of variation; CV = 0.55) than do reptiles (CV = 0.06), birds (CV = 0.38), and mammals (CV = 0.29). This result is consistent with previous findings of high variation in transposable-element content in ray-finned fish genomes (Chalopin et al. 2015; Shao et al. 2019; Marino et al. 2025). We also find that repeat content, driven by transposable elements (Wang et al. 2025), strongly predicts the size of genomes across species (Fig. 2c), consistent with previous findings (Elliott and Gregory 2015; Osmanski et al. 2023).

Next, we address the possibility of ohnologs getting spuriously detected as segmental duplications. Our method for detecting segmental duplications relies on long regions of high sequence similarity and, therefore, generally fails to capture ancient duplicates. Since most species in our dataset have experienced only ancient whole-genome duplication events in their evolutionary history, ohnologs surviving in the species' genomes from such events are expected to lack large stretches of sequence similarity and thus escape detection by our pipeline. For example, the common ancestors of all vertebrates have undergone two rounds of whole-genome duplications (Dehal and Boore 2005), but gene loss, deletions, insertions, and sequence divergence have ensured that few remnants from these ancient events remain detectable as duplicates (Langham et al. 2004; Brunet et al. 2006; Cheng et al. 2018) based on sequence similarity alone. Therefore, with the exception of the brown trout and sterlet sturgeon, which have undergone more recent whole-genome duplications, ohnologs are unlikely to skew our genome-wide analysis of segmental duplications. Thus, no filter was applied to remove ohnologs.

After masking genomes for repeats (including transposable elements) and reasoning that ohnologs are unlikely to bias our results, we called segmental duplications using BISER (Išerić et al. 2022). The percentage of each genome covered by segmental duplications is shown in Fig. 2d. Notably, bird genomes have the smallest segmental-duplication content across taxonomic groups (Fig. 2d), mirroring the small repeat content of their genomes (Fig. 2b). The segmental-duplication content we report in Fig. 2d is generally higher than that reported elsewhere because BISER relies on a segmental-duplication error model that assumes up to 25% sequence dissimilarity rather than the standard 10%. This larger tolerance for dissimilarity allows us to discover older, more diverged segmental duplications that other methods miss.

Interestingly, variation in the segmental-duplication content across genomes is not a predictor of genome-size variation (Fig. 2e). We also note that the repeat content and segmental-duplication content across genomes are uncorrelated (Fig. 2f). If inadequate repeat masking were systematically inflating segmental-duplication calls, we would have observed a negative correlation between repeat content and segmental-duplication content. The lack of such correlation suggests that our repeat masking is adequate.

However, the sterlet sturgeon and the brown trout stand out among ray-finned fishes with unusually large genomes and high apparent coverage by segmental duplications (Fig. 2g). These signals reflect the persistence of ohnologs from relatively recent whole-genome duplications rather than an excess of true segmental duplications, as discussed below.

The sterlet sturgeon (*Acipenser ruthenus*) is a member of the non-teleost “living fossil” sturgeon family. Its lineage has undergone a distinct third round of whole genome duplication (Acipenseriformes-specific third-round or As3R WGD) after divergence from the teleost fish and sometime before 200 million years ago (Redmond et al. 2023). Mirroring its morphological stasis, the sterlet sturgeon's molecular evolution (Krieger and Fuerst 2002; Du et al. 2020; Redmond et al. 2023), in terms of both substitutions and rearrangements, is so remarkably slow that the duplication event was initially misdated to 21.3 million years ago (Cheng et al. 2019). Indeed, the sterlet sturgeon retains an exceptionally high proportion (70%) of duplicated genes from the As3R event (Bi et al. 2021). This combination of ancient whole genome duplication, high gene retention, and slow evolutionary change likely drives the apparent association between segmental duplications and the sterlet sturgeon's large genome size.

The brown trout (*Salmo trutta*), a teleost in the salmonid family, is another notable exception. This species, introduced worldwide as a game fish, is recognized as one of the most invasive fish species globally (Lowe et al. 2000). In addition to the teleost-specific whole-genome duplication (Ts3R WGD), the salmonid family underwent a fourth, salmonid-specific genome duplication (Ss4R) between 50 and 100 million years ago (Alexandrou et al. 2013; Macqueen and Johnston 2014; Lien et al. 2016; Diblasi et al. 2025). This extra round of duplication is associated with larger genome sizes in salmonids (Zhu et al. 2022) and as the only salmonid in our dataset, the brown trout has the largest genome among the ray-finned fish we analyzed. Moreover, ∼10% to 15% of salmonid genomes still exhibit tetrasomic inheritance (Allendorf et al. 2015), promoting gene conversion in these regions (Campbell et al. 2019). These gene conversion events help maintain sequence similarity among duplicated regions, preserving the remnants of the Ss4R event, detectable as spurious segmental duplicates. Thus, the combination of a relatively recent whole-genome duplication and ongoing gene conversion in tetrasomically-inherited regions likely explains the brown trout's large genome and its high fraction of apparent segmental duplications.

### Duplications Close Together and Duplications Far Away

**Fig. 3.**
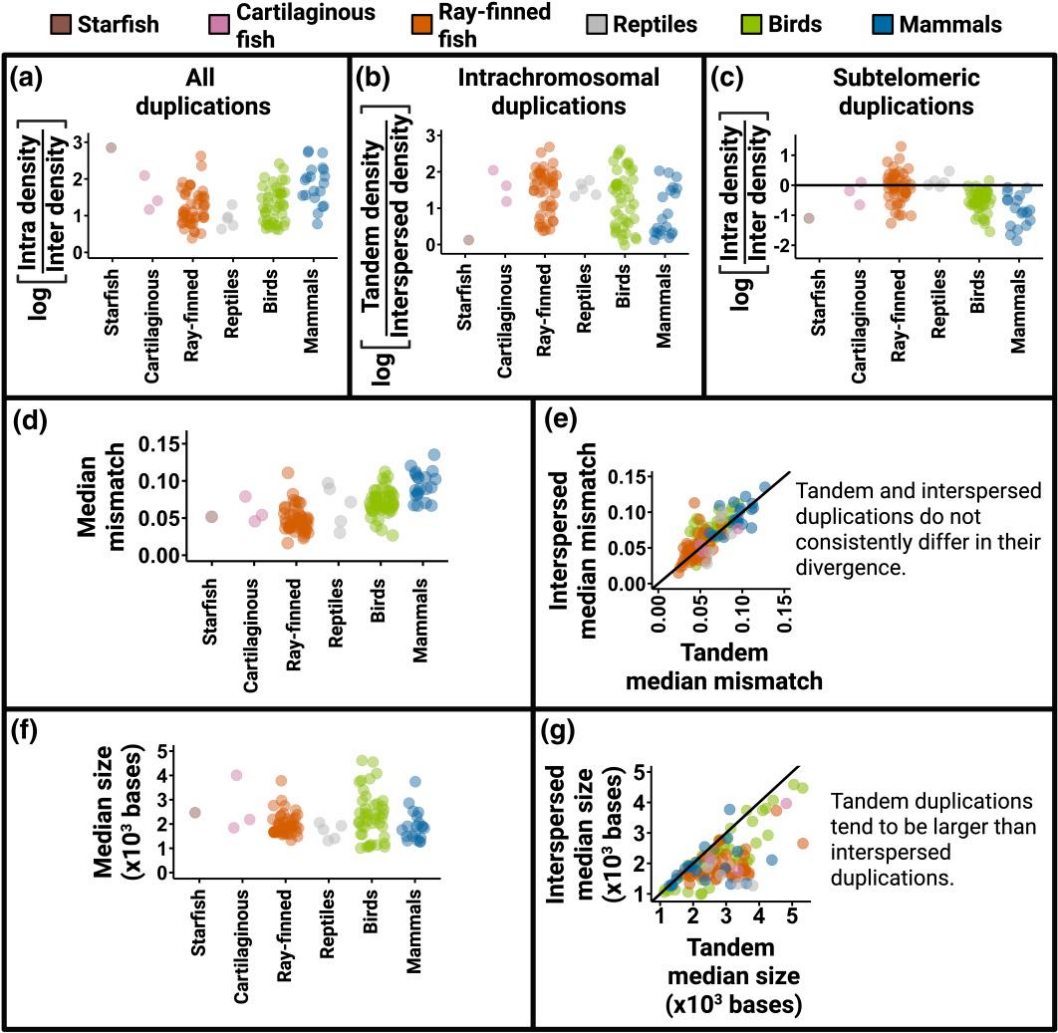
Tandem versus interspersed segmental duplications. a) All vertebrate species show a propensity toward intrachromosomal segmental duplications. The *y* axis is the logarithm (base 10) of intrachromosomal to interchromosomal duplication densities. A *y* axis value > 0 implies intrachromosomal bias. b) Intrachromosomal segmental duplications are biased toward tandem (<1 MB apart) as opposed to interspersed (>1 MB apart) events. The *y* axis is the logarithm (base 10) of the ratio of tandem to interspersed duplication density. A *y* axis value > 0 implies a bias toward tandem duplications. c) Subtelomeric duplications in birds, mammals, and approximately half of ray-finned fish species show a propensity toward interchromosomal events. The *y* axis is the logarithm (base 10) of the ratio of intrachromosomal to interchromosomal subtelomeric duplication density. A *y* axis value > 0 implies an intrachromosomal bias, and a value < 0 implies an interchromosomal bias. d) Distribution of median divergence (mismatch score) between partner duplicates across species in different taxonomic classes. e) Comparison of median mismatch scores between tandem and interspersed duplications. The diagonal line represents equal mismatch scores of tandem and interspersed duplications. There is no consistent difference between tandem and interspersed mismatch scores. f) Distribution of median segmental-duplication size across species in different taxonomic classes. g) Comparison of median duplication sizes between tandem and interspersed duplications. The diagonal line represents equal median sizes of tandem and interspersed duplications. Species to the right of this line tend to have larger tandem duplications relative to interspersed duplications. Most species (110/118) have larger tandem duplications relative to interspersed duplications.

We find that vertebrate segmental duplications are not distributed randomly across genomes. In particular, a genomic region on a given chromosome is more likely to be duplicated on the same chromosome than on a different chromosome (Fig. 3a). This is not to say that every genome contains more intrachromosomal duplications in absolute number. Interchromosomal events are more numerous simply because there are many possible nonhomologous chromosome pairs. However, when corrected for this larger search space, all vertebrates have a higher density for intrachromosomal relative to interchromosomal duplications. Moreover, even within the same chromosome, a region is more likely to be duplicated in tandem (within one million bases; 1 MB) than farther away (Fig. 3b, Fig. S1). One consequence of this higher propensity for tandem duplications is the formation of tandem arrays of related genes (Pan and Zhang 2008).

Interestingly, this density bias toward proximal duplications is reversed near chromosome ends (subtelomeric duplications). Subtelomeric regions in mammals, birds, and about half of the ray-finned fish species show a density bias toward interchromosomal duplications (Fig. 3c). This result mirrors previous findings in humans showing that subtelomeric regions are a pastiche of interchromosomal segmental duplications formed by repeated translocations between chromosome ends, facilitated by nonhomologous end joining (Linardopoulou et al. 2005).

These spatial patterns suggest that two broad mechanisms shape duplication landscapes. Local errors in recombination and DNA replication create a propensity toward tandem duplications, whereas nonhomologous end joining creates a subtelomeric propensity toward interchromosomal duplications.

Finally, we examined how the size (alignment span) and sequence divergence (mismatch score) of duplications differ between tandem (<1 MB apart) and interspersed (>1 MB apart or on different chromosomes) duplicates. We find that the median divergence between duplicates is low (Fig. 3d; Fig. S2), and there is no consistent difference in divergence between tandem and interspersed duplications across species (Fig. 3e).

More interestingly, we observe that while most duplications are small, on the order of 10^3^ base pairs (Fig. 3f; Fig. S3), tandem duplications tend to be larger than interspersed ones (Fig. 3g). This size difference is consistent with observations in humans (Zhang et al. 2005), mice (Francoeur et al. 2025), and even Drosophila (Fiston-Lavier et al. 2007), suggesting that it may be a general feature of animal genomes. This result may also explain the finding in mammals that interchromosomal daughter gene copies evolve faster relative to their parent paralog than do intrachromosomal daughter copies (Moffett et al. 2025). Because interchromosomal duplicates are smaller, they are less likely to include the parent gene's cis-regulatory elements, leaving the parent constrained and the daughter free to diverge; by contrast, larger intrachromosomal duplicates more often retain regulatory sequences, so both copies begin under similar constraint.

### Rapidly Evolving Segmental-duplication Landscapes

To quantify segmental-duplication landscapes, we constructed a separate segmental-duplication network for each of the 118 species in our dataset. Here, nodes represent genomic regions and edges represent duplications. Edge weights indicate similarity scores, representing how recently the duplication emerged. For each species, we calculated 13 network properties and the average similarity score across duplications (a non-network property). We sought to use these 14 metrics collectively to quantify the segmental-duplication landscape for that species (see Methods).

To assess whether the metrics quantifying segmental-duplication landscapes were influenced by assembly qualities, we calculated correlations between each of the 14 landscape metrics and four assembly quality indicators: coverage width, contig NG50, scaffold NG50, and percentage of the assembly composed of unplaced contigs. Only 4 of the 14 metrics (network density, weighted network density, *β*, and mean similarity score) were moderately correlated with at least one assembly quality metric (0.3 ≤ |r| ≤ 0.5 and raw *P* < 0.01). Thus, 10 out of the 14 metrics of segmental-duplication landscapes are largely independent of assembly quality (Figs. S4 to S7).

**Fig. 4.**
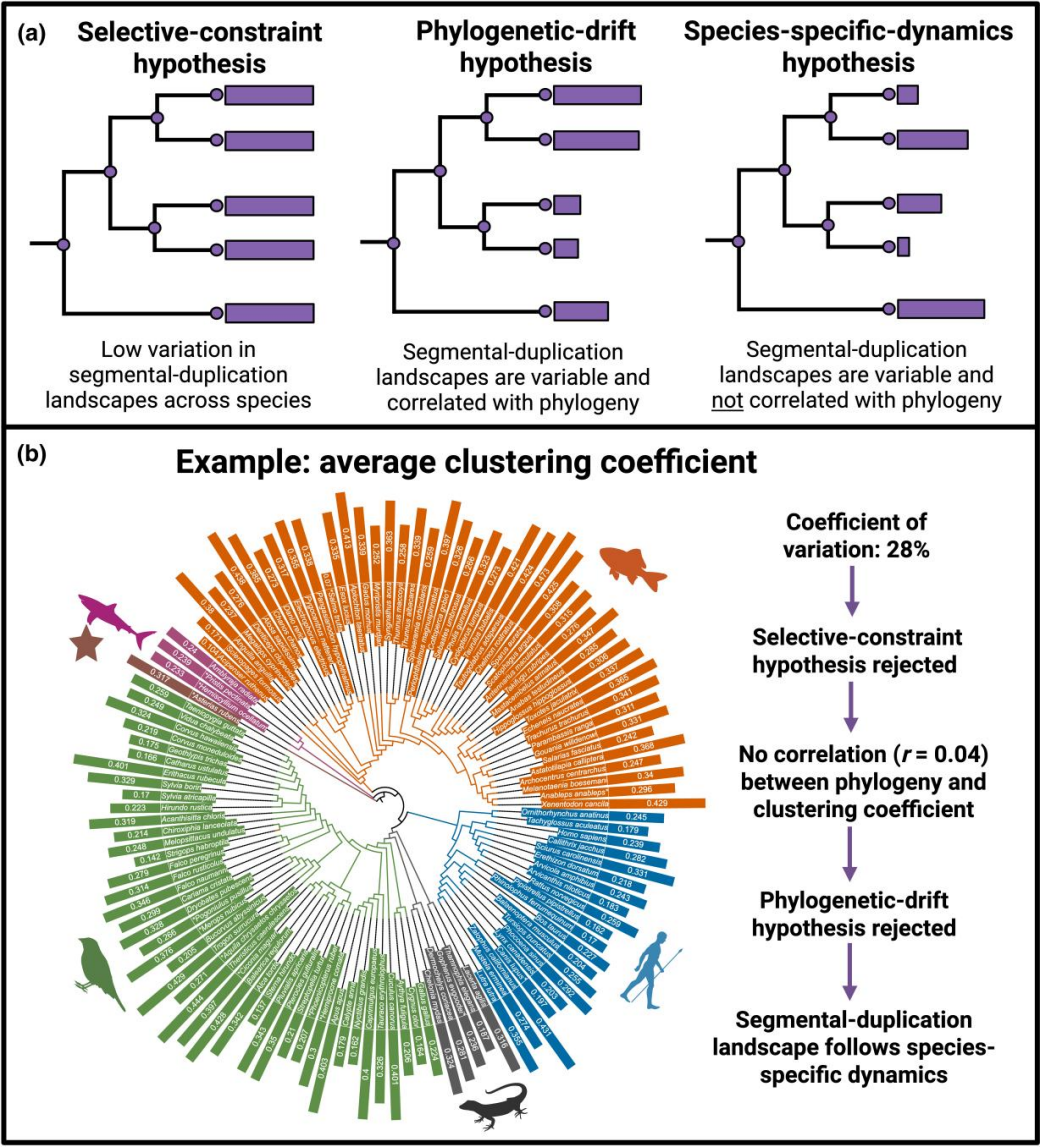
Hypotheses for the evolution of segmental-duplication landscapes. a) Expectation for variation in segmental-duplication landscapes under each hypothesis. The leaves in the trees in each schematic represent species. The length of the purple bar represents the value of a hypothetical metric of the segmental-duplication landscape for the corresponding species. The figure shows the expected variation in this metric under three hypotheses. b) A phylogenetic tree of 118 species annotated with the average clustering coefficient values. The height of the bar on each species indicates the value of the average clustering coefficient. Species' names appended with an asterisk mean that a close substitute for the species was used to generate the tree (see Table S2). The variation in the average clustering coefficient across species matches the expectation under the species-specific-dynamics hypothesis.

Next, we use the 10 landscape metrics that are largely independent of assembly quality to study the evolution of segmental-duplication landscapes across vertebrates. Specifically, we tested three hypotheses: (1) the “selective-constraint hypothesis,” which states that the segmental-duplication landscape is highly conserved across species, showing minimal variation; (2) the “phylogenetic drift hypothesis,” which states that segmental-duplication landscapes differ between species in accordance with their phylogenetic distance; and (3) the “species-specific-dynamics hypothesis” stating that the segmental-duplication landscape evolves so rapidly that it does not strongly correlate with phylogeny (Fig. 4a). We note that these three hypotheses are not mutually exclusive, but it is possible to parse which of these three is most dominant in shaping segmental-duplication landscapes.

To test the selective-constraint hypothesis (that evolutionary pressure conserves segmental-duplication landscapes across species), we analyzed all 10 assembly-quality-independent metrics across species. Following previous studies (Al-Sallami et al. 2014; Kumar et al. 2018), we considered a metric's variation across species to be low if its coefficient of variation across species was <10%. We found that none of the segmental-duplication-landscape metrics showed low variation (Table S1), suggesting that segmental-duplication landscapes are not conserved in vertebrates. In fact, four out of the ten metrics exhibit coefficients of variation exceeding 100%. Based on these findings, we reject the selective-constraint hypothesis and shift focus to the two alternative hypotheses (ie phylogenetic drift and species-specific-dynamics) to explain the observed variation in segmental-duplication landscapes.

To differentiate between the phylogenetic-drift hypothesis and the species-specific-dynamics hypothesis, we calculated pairwise distances for each of the ten metrics across species pairs. We correlated these with phylogenetic distances obtained from TimeTree (see Methods) (Kumar et al. 2022). If a species was not available in TimeTree, we used a closely related substitute (Table S2). If most of these 10 metrics correlate strongly with phylogeny, we favor the phylogenetic-drift hypothesis; otherwise, we favor the species-specific-dynamics hypothesis.

Firstly, we found that none of the ten metrics of segmental-duplication landscapes correlates strongly (|r|>0.3; see Methods for justification of the threshold) with phylogeny across vertebrates (Table S3). This lack of strong correlation led us to reject the phylogenetic-drift hypothesis in favor of the species-specific-dynamics hypothesis. We demonstrate this reasoning using the average clustering coefficient, one of the ten metrics, in Fig. 4b. We note, however, that five of the ten assembly-quality-independent metrics do show weak correlation (0.1<|r|<0.3) with phylogeny. We do not consider this weak correlation to constitute strong evidence in favor of the phylogenetic-drift hypothesis. Secondly, even the few closely related species pairs in our dataset (time since divergence < 50 million years) do not have more similar segmental-duplication landscapes than do more distantly related species pairs (Cohen's |*d*| < 0.2 across metrics; Table S4; Fig. S8). Lastly, restricting the analysis only within mammals, we find that four landscape metrics correlate with phylogeny. Nevertheless, this result is driven by differences in assembly quality between monotremes and therian mammals (Fig. S9).

Overall, our findings suggest that the segmental-duplication landscapes are rapidly evolving.

These results likely stem from sparse sampling within clades, with segmental-duplication landscapes being governed by recent clade-specific bursts of segmental duplications. Indeed, such a segmental-duplication burst was reported to occur in the ancestor of the African great apes (Marques-Bonet et al. 2009).

We note that one previous study (Abdullaev et al. 2021) reported that segmental-duplication landscapes follow the phylogenetic-drift hypothesis instead of the species-specific-dynamics hypothesis supported by our results. However, their conclusion was based on only nine species and one network property (component size distribution). With our large dataset of 118 species, we found that the component size distribution also follows species-specific-dynamics, consistent with the results based on our 10 landscape metrics (see Text S1). This result suggests that the results of Abdullaev et al. (2021) may be limited in generalizability due to restricted species sampling and focus on a single network property.

### Functional Enrichments

We conducted gene ontology (GO)-enrichment analyses using g:Profiler for 11 species represented in both our dataset and g:Profiler annotations. These 11 species included 4 mammals (human, platypus, dolphin, and greater horseshoe bat), 4 ray-finned fishes (eastern happy, brown trout, lumpfish, and climbing perch), 2 birds (kakapo and golden eagle), and 1 reptile (Good's thornscrub tortoise). For each species, we identified the top 100 genes with the largest number of overlapping segmental duplications and tested these sets for enrichment of biological processes.

We did not detect any biological processes enriched across multiple species, suggesting that the types of genes amplified by segmental duplications are largely lineage-specific. Nonetheless, several species showed lineage-specific enrichments, including response to pheromones (*P* value adjusted for multiple hypothesis testing, $P_{\mathrm{adj}}$ = 1.1 × 10^−11^) and sensory perception of chemical stimulus ($P_{\mathrm{adj}}\;$ = 2.7 × 10^−8^) in platypus, regulation of DNA-templated transcription ($P_{\mathrm{adj}}\;$ = 6.5 × 10^−11^) in humans, synaptic signaling ($P_{\mathrm{adj}}\;$ = 0.0011) in greater horseshoe bat, protein metabolic process ($P_{\mathrm{adj}}\;$ = 0.039) in dolphin, multicellular organismal process ($P_{\mathrm{adj}}$ = 0.049) in brown trout, and regulation of lymphangiogenesis ($P_{\mathrm{adj}}$ = 0.049) in lumpfish.

**Fig. 5.**
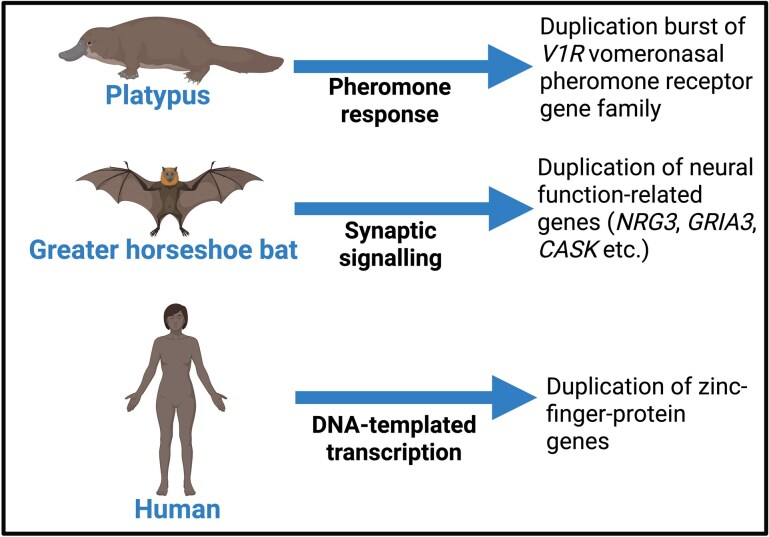
Functional enrichments of hyper-duplicated genes in the platypus, the greater horseshoe bat, and humans.

The case of the platypus is particularly striking: the enrichment is driven by the expansion of the vomeronasal pheromone receptor *V1R* gene family, consistent with previous findings (Grus et al. 2007; Zhou et al. 2021). Similarly, the functional enrichment in the greater horseshoe bat is driven by duplication of neural function-related genes, while in humans, the functional enrichment of transcription regulation is driven by the expansion of zinc-finger-protein genes (Fig. 5).

## Discussion

In this study, we generate segmental-duplication calls from repeat-masked long-read-sequenced genomes of 117 vertebrate species and one starfish. We caution that like most pipelines for detecting segmental duplications, our approach is sensitive to repeat masking. While we have used the most comprehensive repeat libraries available to mask genomes and indirectly show in our analysis that repeat masking is adequate, minor effects of inconsistencies in repeat masking across species may nonetheless linger. Indeed, the quality of repeat masking is expected to improve as lineage-specific transposable elements become better characterized.

Based on these segmental-duplication calls, we find that vertebrate genomes exhibit a higher propensity for tandem duplications relative to interspersed duplications, mirroring previous findings in humans. However, in subtelomeric regions, birds and mammals show the opposite propensity toward interspersed duplications. Moreover, we find that tandem duplications tend to be larger than interspersed duplications in vertebrates.

We quantified segmental-duplication landscapes based on metrics extracted from segmental-duplication networks. Comparing the differences in these properties against phylogenetic distances between species, we find that vertebrate segmental-duplication landscapes are rapidly evolving. The rapid evolution of segmental-duplication landscapes across species may confer unique adaptive potentials, enabling certain lineages to respond more effectively to selective pressures. This may, in part, explain why some lineages are virtual living fossils while others are more prone to undergoing adaptive radiation. This result adds to the growing recognition (Wagner 2008; Dennis and Eichler 2016; Pajic et al. 2019; Mérot et al. 2020; Aqil et al. 2023) of the importance of structural variation in evolution.

Finally, we show that hyperduplicated genes in certain species are enriched for specific biological processes. For example, in the platypus, these genes are enriched for pheromone response, driven by expansion of the vomeronasal pheromone receptor *V1R* gene family. Such expansions highlight how segmental duplications can become molecular substrates for evolutionary novelty.

The strength of our study is the application of network-based methods to study genomic structure. Network properties capture relationships among duplicated genomic regions that are invisible to traditional analyses, offering deeper systems-level insights into the structural organization of genomes.

Future work could extend our network framework to multilayer segmental-duplication networks, where each species represents a layer. Here, interspecies edges can connect orthologous genomic regions between species. This fine-grained approach, analogous to multilayer coexpression networks (Russell et al. 2023), would enable the identification of communities of duplicated genomic regions that persist across layers (species). It would allow for the systematic discovery of gene copy number expansions underlying both lineage-specific adaptation and convergent evolution. A practical starting point could focus on a smaller and more closely related group of species such as primates, rodents, or carnivores, for which genomic alignments are more reliable. Overall, although analyzing multilayer segmental-duplication networks would be computationally demanding, it would provide deeper comparative insights than analyses limited to metrics derived from single-species segmental-duplication networks.

As long-read sequencing technologies continue to improve and become commonplace, network-based approaches will become even more powerful tools for characterizing complex genomic phenomena. Overall, our study highlights how integrating network analysis with evolutionary biology can provide insights into genome biology.

## Methods

### Data

We downloaded FASTA files for all 150 curated assemblies from GenomeArk (https://www.genomeark.org) as of August 16th, 2022. GenomeArk holds genome sequences generated by the Vertebrate Genomes Project (Formenti et al. 2026). These assemblies included sex chromosomes. In this study, sex chromosomes and autosomes were analyzed together. For humans, we used the hg38 reference assembly for better comparison to previous studies on duplications in humans.

Before looking for segmental duplications (SDs), i.e. duplications with a size of at least one kilobase, it is necessary to mask all short repeats within each genome as much as possible to avoid false SD positives and improve the running time of SD detection software. This is because BISER (Išerić et al. 2022) (the SD detection software we use) assumes that all short repeats are filtered out; if they are not, short tandem repeat clusters and transposable elements will be called as SDs despite not being biologically so. Indeed, if these repeats are not masked, short repeats or transposable elements may masquerade as segmental duplications. We used RepeatMasker (v4.1.3) for this purpose. Since masking was inadequate for many non-mammalian species using the default masking library (Dfam minimal), we employed the complete Dfam 3.6 library. Additionally, we augmented this masking using species-specific repeat libraries from RepBase (v10/26/2018). Indeed, for all mammals, all birds, all reptiles, half of the ray-finned species, and 2 out of 3 of cartilaginous fish species, we used species-specific libraries. For the starfish, one cartilaginous fish, and the other half of the ray-finned fishes, species-specific libraries were not available. So, we used class-level repeat libraries in addition to the complete Dfam library to mask them. Thus, the repeat masking is not expected to be highly inconsistent across taxa. Masking took from a few hours to a few weeks (it was especially performance-intensive on *Actinopterygii* genomes) and masked on average 25% of each genome: from 2% (*Asteroidea*) to 82% (*Bal. musculus*). We note that at the time of our analysis (data access date August 16th, 2022), the starfish genome was the only invertebrate genome available in the GenomeArk database.

Once the genomes were masked, we used BISER v1.2 (Išerić et al. 2022) to find all SDs within them. BISER relies on the SD error model (Numanagic et al. 2018) that assumes that (1) sequence similarity of SDs is higher than 75% (in other words, the error is below 25%), (2) random point mutations account for 15% of the total error and that they follow Poisson error distribution and are independent of each other (Jain et al. 2020); and (3) large indels and block variants account for the remaining 10% of the total error. The higher error rate threshold enables BISER to detect older SDs that occurred before the primate split in evolutionary history [the standard cutoff error threshold of 10% (Bailey and Eichler 2006) is sufficient only for detecting primate SDs (Numanagic et al. 2018)].

BISER finds all SDs in a given genome using a *k*-mer-based sketch of Jaccard distance through MinHash (Broder 1997) and *k*-mer windowing (Jain et al. 2020) to approximate edit distance under the described SD error model. This model allows it to quickly calculate SDs in the whole genome and decompose them into core blocks (Jiang et al. 2007). However, like all SD detection tools, BISER is extremely sensitive to the repeat masking quality; in our runs, it took from 4 min (good quality masking) to more than 10 d (low-quality masking; median time: 10 min) to find all SDs in a given genome. Our pipeline ran successfully for 118 genomes. All analysis in this manuscript is based on those 118 genomes. About 109 of the 118 assemblies are pseudo-haploid consensus-collapsed assemblies. The remaining nine are true (maternal or paternal) haploid assemblies obtained from trio-binning.

### Evolutionary Distances

To get a phylogenetic tree for the 118 species analyzed here (Fig. 1a), we used a list of Latin binomials corresponding to these species and used it as input for TimeTree 5 (Kumar et al. 2022). On the tree, 14 species were replaced by close substitutes (Table S2). For our analysis, we used these close substitutes as proxies for the original species to measure phylogenetic distances. When using substitutes, we still use the names of the original species and append them with an asterisk. The output from TimeTree was a tree file in the Newick format with branch lengths in terms of the number of years. We used the cophenetic.phylo() function from the “ape” library in R (Paradis and Schliep 2019) to obtain evolutionary distances (in years) between each pair of species. The tree was visualized using the interactive Tree of Life (Letunic and Bork 2021).

### Network Construction

**Fig. 6.**
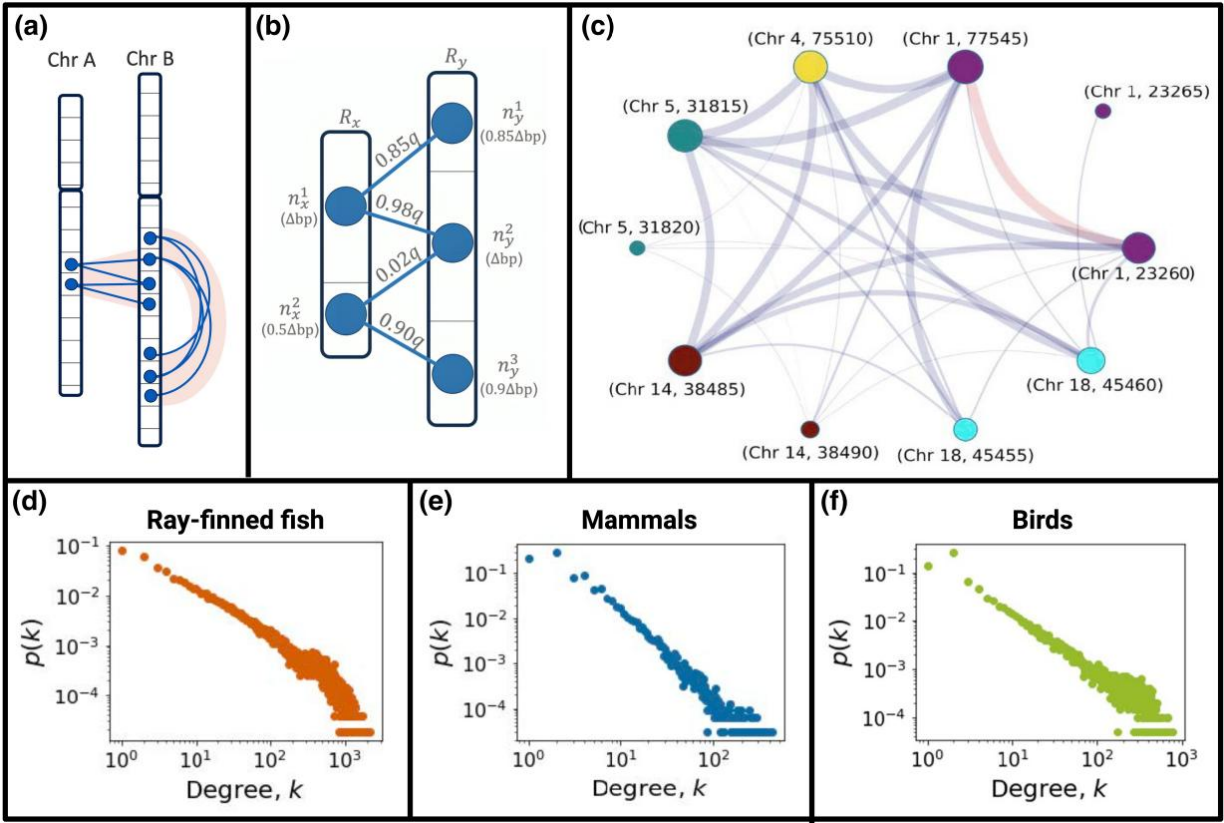
Network construction and degree distribution. a) Schematic for edge construction between two duplicated regions. b) Examples of edges for two chromosomes. The part of the network shown here is a magnification of edges between Chr A and Chr B in a). c) A part of the weighted segmental-duplication network constructed for *Homo sapiens*. Different node colors represent different chromosomes. The number followed by the comma represents the starting coordinate (kbp) of the node on the chromosome d–f. The degree distribution of the segmental-duplication network from a representative species of ray-finned fish, mammals, and birds, respectively.

We construct a weighted network for each species from the data described above. We construct the network by considering consecutive windows, sized Δ= 5,000 base pairs (5 kbp) on chromosomes (Fig. 6a). We use each such window as a node and connect pairs of nodes by weighted edges. We determine weighted edges as follows. In general, the duplication size does not coincide with Δ or multiples of it. For instance, assume that a region $R_{x}$ of length 1.5Δ bp is duplicated in another region $R_{y}$ of length 2.75Δ bp and that the similarity score between them is *q*. We assume that the starting coordinate of the region $R_{x}$ corresponds to the starting coordinate of region $R_{y}$ and the same for the ending coordinate. Therefore, a window of size Δ in $R_{x}$ corresponds to a subregion of $R_{y}$ of size 2.75Δ×(1Δ/1.5Δ)=1.83Δ bp. For expository purposes, we also assume that $R_{x}$ is contained in two nodes, $n_{1}^{x}$ of size 1Δ bp and $n_{2}^{x}$ of size 0.5Δ bp, and that $R_{y}$ is contained in three nodes, $n_{1}^{y}\;$ of size 0.85Δ bp, $n_{2}^{y}\;$ of size 1Δ bp, and $n_{3}^{y}\;$ of size 0.9Δ bp (Fig. 6b). We calculate the edges, and their weights as follows. We assume that the size of $R_{y}$ is larger than $R_{x}$ without loss of generality. First, we find the subregions of $R_{y}$ that overlap the projection of the subregion of node $n_{i}^{x}$ on $R_{y}$, for each *i* th node contained in $R_{x}$. This procedure determines the nodes in $R_{y}$ that are adjacent to $n_{i}^{x}$ by an edge. For example, in Fig. 6b, node $n_{1}^{x}\;$ is projected to the first 1.83Δ bp of $R_{y}$. Therefore, we connect $n_{1}^{x}\;$ to $n_{1}^{y}\;$ and $n_{2}^{y}\;$ by an edge. Note that the entirety of $n_{1}^{y}\;$(of size 0.85Δ bp) overlaps with the projection of $n_{1}^{x}\;$ and that the first 0.98Δ bp of $n_{2}^{y}\;$ overlaps with the projection of $n_{1}^{x}$. Likewise, $n_{2}^{x}$ is projected to the last 0.92Δ bp of $R_{y}$. Because $n_{3}^{y}$ accounts for the last 0.9Δ bp of $R_{y}$, we connect $n_{2}^{x}$ to each of $n_{2}^{y}$ and $n_{3}^{y}$ by an edge. Note that the last 0.02Δ bp of $n_{2}^{y}\;$ overlaps with the projection of $n_{2}^{x}$ and that the entirety of $n_{3}^{y}$ (of size 0.9Δ bp) overlaps with the projection of $n_{2}^{x}$. In this manner, we define an edge between a node in $R_{x}$ and a node in $R_{y}$ if and only if there is a positive overlap between $R_{y}$ and the projection of $R_{x}$ on $R_{y}$. Second, we define the weight of the edge between $n_{i}^{x}$ and $n_{j}^{y}$ by


(1) $$w_{i,j}^{x,y}\;\;=\frac{\begin{array}{c} q\;\times (\mathrm{size}\;\mathrm{of}\;\mathrm{part}\;\mathrm{of}\;n_{j}^{y}\;\mathrm{covered}\;\\ \mathrm{by}\;\mathrm{the}\;\mathrm{projection}\;\mathrm{of}\;n_{i}^{x}\;\mathrm{on}\;R_{y}) \end{array}}{\Delta }$$

The generated networks are undirected. We allow a node to participate in edges with multiple duplications and edges with other nodes on the same or different chromosomes, as shown in Fig. 6a and b. We show a part of the network of *Homo sapiens* in Fig. 6c. The first component of each node label represents the chromosome number, and the second component represents the starting coordinate of the node in kbp. We recall that the node spans from the starting position to the subsequent 5 kbp. For example, node (Chr 1, 23260) is located on chromosome 1 and spans from 23,260 kbp to 23,264.999 kbp. We also show the degree distribution of three representative (chosen arbitrarily) networks from three taxonomic classes: ray-finned fish, mammals, and birds in Fig. 6d, e, and f, respectively.

### Network Properties

To understand the underlying duplication patterns, their evolutionary significance, and their potential impact on genomic architecture across species, we computed the following network properties for each species.

We denote the adjacency matrix of the segmental-duplication network by $A=(a_{ij})$, where the nodes are the genomic regions of size 5 kbp. The adjacency matrix represents whether each node pair is connected by an edge. We use the duplication similarity score as the weight of the edge between the *i*th and *j*th nodes, which we denote $w_{ij}$. Note that $a_{ij}=a_{\;ji}=\;1$ if $w_{ij}=\;w_{\;ji}>0$, $a_{ij}=a_{\;ji}=0$ if $w_{ij}=w_{\;ji}=0$, and by assumption that $a_{ii}=w_{ii}=0$. The degree of the *i* th node is defined by


(2) $$k_{i}=\overset{N}{\underset{\;j=1;j\neq i}{\sum }}\;a_{ij},$$

where *N* is the number of nodes in the network. The degree represents the number of other nodes that the focal node is adjacent to by an edge. A high-degree node is a region that has been duplicated many times and, therefore, is suggested to be a hotspot of duplication activity.

The weighted degree, also called the node strength (Barrat et al. 2004), of the *i*th node is given by


(3) $$s_{i}=\overset{N}{\underset{\;j=1;j\neq i}{\sum }}\;w_{ij}.$$

The node strength measures the cumulative similarity of all duplications for a focal genomic region across the genome. A node (region) with high strength is a region that has been duplicated many times and, on average, has high sequence similarity with its duplicates.

We have explained network metrics that are calculated for each node. We now explain other network metrics that are measured for each segmental-duplication network, or equivalently, for each species. Some metrics use the node degree or the weighted degree of all the nodes in the segmental-duplication network.

The mean degree is given by


(4) $$\bar{k}=\frac{1}{N}\overset{N}{\underset{i=1}{\sum }}\;k_{i}=\frac{1}{N}\overset{N}{\underset{i=1}{\sum }}\;\overset{N}{\underset{\;j=1}{\sum }}\;a_{ij}.$$

It is the average of the node degrees over all the nodes. It represents the average number of duplication events per genomic region. A higher mean degree indicates a more densely interconnected duplication network, possibly reflecting extensive segmental-duplication events.

The mean node strength is given by


(5) $$\bar{s}\;=\frac{1}{N}\overset{N}{\underset{i=1}{\sum }}\;s_{i}=\frac{1}{N}\overset{N}{\underset{i=1}{\sum }}\;\overset{N}{\underset{\;j=1}{\sum }}\;w_{ij}.$$

It is a measure of the duplication similarity score per pair of genomic regions. Larger $\bar{s}$ values suggest stronger duplication relationships between genomic regions overall in the entire network.

We measure the heterogeneity of a distribution by the coefficient of variation (CV). The CV of the degree distribution and that of the strength distribution is given by


(6) $$CV_{k}=\frac{\sigma_{k}}{\bar{k}}$$

and


(7) $$CV_{s}=\frac{\sigma_{s}}{\bar{s}},$$

respectively, where $\sigma_{k}$ and $\sigma_{s}$ are the sample SDs of the node's degree and strength, respectively. The CV of the degree distribution measures how much variation across the *N* nodes exists in terms of the number of duplication connections a node has. A large $CV_{k}$ value indicates that some regions have markedly high degrees, representing duplication hubs, while other regions have small degrees. A small $CV_{k}$ value suggests a relatively uniform duplication pattern across genomic regions. The CV of the node strength distribution measures the variation across the *N* nodes in terms of the cumulative duplication similarity score for a node. A large $CV_{s}$ value suggests that some genomic regions have markedly strong duplication relationships with other regions. A small $CV_{s}$ value indicates that duplication similarities are more evenly distributed across the genome.

The density of the network is defined by


(8) $$\rho =\frac{1}{N(N-1)}\overset{N}{\underset{i=1}{\sum }}\;\overset{N}{\underset{\;j=1}{\sum }}\;a_{ij}$$

and represents the fraction of node pairs connected by an edge (Wasserman et al. 1994; Newman 2018). High density means that segmental duplications are widespread, with many genomic region pairs sharing duplications. Low density suggests that duplications occur in a more isolated manner, possibly reflecting evolutionary constraints.

The weighted density of the network is given by


(9) $$D=\frac{1}{N(N-1)}\overset{N}{\underset{i=1}{\sum }}\;\overset{N}{\underset{\;j=1}{\sum }}\;w_{ij}$$

and represents the average edge weight over all node pairs, including those that are not adjacent by an edge (Wasserman et al. 1994). The weighted density reflects the overall duplication similarity across the genome. A large *D* value suggests that duplications tend to be strong in terms of the similarity score and widespread. A small *D* value suggests that duplications are weaker or sparser.

The average clustering coefficient (Watts and Strogatz 1998) is defined by


(10) $$C\;=\;\frac{1}{N}\underset{i\in G}{\sum }\;C_{i},$$

where $C_{i}$ is the local clustering coefficient for the *i* th node given by


(11) $$C_{i}=\frac{\begin{array}{c} 2\times (\mathrm{Number}\;\mathrm{of}\;\mathrm{pairs}\;\mathrm{of}\;\mathrm{neighbors}\;\mathrm{of}\;\\ \mathrm{node}\;i\;\mathrm{that}\;\mathrm{are}\;\mathrm{adjacent}\;) \end{array}}{k_{i\;}(k_{i}-1)}.\;$$

The average clustering coefficient represents the propensity that two neighbors of a node are also directly connected. A high *C* value suggests that duplications tend to form connected clusters in the sense of triangles, possibly indicating duplication hotspots. A low *C* value suggests that duplications occur in a more random fashion.

In addition to these properties, we also measure the mean, CV, and skewness of the edge weight, $w_{ij}$ (Watts and Strogatz 1998). The mean edge weight represents the average similarity score between duplicated regions per duplication event. A high mean edge weight suggests recent or highly conserved duplications. A low mean weight suggests that duplications are generally divergent, suggesting older duplication events or functional divergence. The CV of the edge weight measures variability in duplication similarity across the duplicated region pairs. A large value of the CV of the edge weight implies a mix of old and new duplications. A small value of the CV of the edge weight suggests that the duplication similarity is relatively uniform across duplicated region pairs, indicating consistent duplication mechanisms for different duplicated region pairs. The skewness for the edge weight is given by


(12) $$\gamma \;=\;\frac{\frac{1}{2N}\;\sum_{i=1}^{N}\;\sum_{\;j=1}^{N}\;A_{ij}{\left(w_{ij}-\frac{\bar{s}}{\bar{k}}\right)}^{3}}{\sigma_{w}^{3}},$$

where $\sigma_{w}$ is the sample SD of the edge weight. The skewness of the edge weight measures the asymmetry in the distribution of the duplication similarity score (ie edge weight). Positive skewness (γ>0) suggests that most duplication events have low similarity, with a few having exceptionally high similarity. Negative skewness (γ<0) suggests that most duplications have high similarity, which may indicate genome regions with conserved segmental duplications. It should be noted that the CV also measures the extent of variation in the duplication similarity score; however, unlike the skewness, the CV does not measure the asymmetry.

We quantify the abundance and strength of cis-duplications relative to those of transduplications by measuring the so-called density ratio. To compute the density ratio for a given chromosome to other chromosomes, we first consider the density, $\rho_{\mathrm{cis}}$, of the subnetwork that consists of the edges that connect two nodes in the same chromosome. Second, we compute the density, $\rho_{\mathrm{trans}}$, of the subnetwork consisting of the edges connecting the node in the given chromosome to other chromosomes. Then, we compute the density ratio $\frac{\rho_{\mathrm{cis}}}{\rho_{\mathrm{trans}}}$ for the given chromosome. Finally, we calculate the mean of the density ratios over all chromosomes for a given species. We measure density ratios for both the unweighted network and the weighted network. A high unweighted density ratio implies that duplications mostly occur within the same chromosome. This case suggests localized duplication mechanisms. A low-unweighted density ratio implies that interchromosomal duplications are relatively frequent, which could be due to chromosomal translocations, rearrangements, or interspersed duplications. A high-weighted density ratio signifies that intrachromosomal duplications exhibit stronger similarity scores than interchromosomal ones overall. This case implies that intrachromosomal duplications are either more conserved or more abundant than interchromosomal ones. A low-weighted density ratio suggests that interchromosomal duplications have strong average similarity or are abundant.

We also explore how the node strength depends on the node degree for each network in a potentially nonlinear manner. We fit the following relation (Barrat et al. 2004):


(13) $$\ln\;\bar{s}=\alpha +\beta \;\ln\;k,$$

where *k* is the node's degree and $\bar{s}$ is the node strength averaged over all the nodes whose degree is *k*. To estimate *α* and *β*, we fitted a line to the set of points $\left\{\ln\;k,\ln\;\bar{s}\right\}$ using sklearn (Pedregosa et al. 2011) in Python. Equation (13) implies that $\bar{s}$ is proportional to $k^{\beta }$. If β>1, then hubs, ie large-degree nodes, tend to have stronger duplications, implying that duplication hubs preferentially duplicate with highly similar regions. If β=1, there is no such correlation between the node's degree and edge weight, implying that the duplication strength is independent of how many duplications a node has experienced. Various empirical weighted networks yield β>1 (Barrat et al. 2004).

To calculate the network properties, we use NetworkX version 2.8.5 in Python (Hagberg et al. 2008).

### Comparison with Phylogenetic Tree

We calculate all the 14 properties for each species in the dataset. For a given property, which is a scalar, we quantify the dissimilarity between two species *p* and $p^{\prime }$ in terms of the property by


(14) $$d_{\Xi }(\;p,p^{\prime })\;=\;|\Xi_{p}-\Xi_{\;p^{\prime }}|,$$

where $\Xi_{p}$ and $\Xi_{\;p^{\prime }}$ represent the network property for species *p* and $p^{\prime }$, respectively. We compare $d_{\Xi }(\;p,p^{\prime })$ to the phylogenetic distance between *p* and $p^{\prime }$ to explore which network-based measure may be associated with the phylogeny. We measure the correlation between the phylogenetic distance and $d_{\Xi }(\;p,p^{\prime })$ by regarding each pair of species as a sample.

Here, we note an important concern. Our correlation analysis involves 6,962 species pairs (118 × 117/2). It is well recognized in statistics that with such a large number of data points, hypothesis testing could yield “significant” results even for trivial effects (Berkson 1938; Cohen 1994). This phenomenon (“crud factor”) (Clayton 2021) cautions against overinterpreting *P*-values in large datasets. Therefore, rather than relying solely on *P*-value thresholds, we consider the two variables to be strongly correlated only if |r|>0.3, the conventional threshold for medium effect size (Cohen 1988) that is routinely used in biology (Fredlund et al. 2012; Friedman and Alm 2012; Pai et al. 2019; Polk et al. 2025).

For the comparison in taxonomic class level, we omitted the analysis of the starfish, cartilaginous fish, and reptile classes because each of them had at most five species.

## Supplementary Material

evag043_Supplementary_Data

## Data Availability

The segmental-duplication calls obtained using BISER, for the 118 genomes analyzed in this study have been deposited on FigShare. In addition to the files containing segmental-duplication calls, the file “Species_and_Assembly_Info_Final.csv,” containing taxonomic details of species, genome sizes, qualities of assembly, and the link to download the relevant FASTA (genomic sequence) files, is also available on FigShare. The FigShare repository also contains the file “Final_Measures_of_SD_Landscape.csv” containing the 14 segmental-duplication-landscape metrics for the 118 species (117 vertebrates and one starfish species) analyzed in this study. This data can be accessed using the following link:https://figshare.com/articles/dataset/Segmental_Duplication_Calls_BISER_Vertebrates_LongReadGenomes/27859545. The code to generate segmental-duplication calls using BISER can be found on GitHub (https://github.com/0xTCG/biser).
